# Testing Dynamic Balance in People with Multiple Sclerosis: A Correlational Study between Standard Posturography and Robotic-Assistive Device

**DOI:** 10.3390/s24113325

**Published:** 2024-05-23

**Authors:** Jessica Podda, Giorgia Marchesi, Alice Bellosta, Valentina Squeri, Alice De Luca, Ludovico Pedullà, Andrea Tacchino, Giampaolo Brichetto

**Affiliations:** 1 Italian Multiple Sclerosis Foundation, 16149 Genoa, Italy; jessica.podda@aism.it (J.P.); ludovico.pedulla@aism.it (L.P.); giampaolo.brichetto@aism.it (G.B.); 2 Movendo Technology S.R.L, 16149 Genoa, Italy; giorgia.marchesi@movendo.technology (G.M.); valentina.squeri@iit.it (V.S.); alice.deluca@iit.it (A.D.L.); 3 Department of Experimental Medicine, University of Genoa, 16126 Genoa, Italy; alice.bellosta@edu.unige.it; 4 AISM Rehabilitation Service, 16149 Genoa, Italy

**Keywords:** dynamic balance, robotic assessment, hunova^®^, EquiTest^®^, posturography, Multiple Sclerosis

## Abstract

Background: Robotic devices are known to provide pivotal parameters to assess motor functions in Multiple Sclerosis (MS) as dynamic balance. However, there is still a lack of validation studies comparing innovative technologies with standard solutions. Thus, this study’s aim was to compare the postural assessment of fifty people with MS (PwMS) during dynamic tasks performed with the gold standard EquiTest^®^ and the robotic platform hunova^®^, using Center of Pressure (COP)-related parameters and global balance indexes. Methods: Pearson’s ρ correlations were run for each COP-related measure and the global balance index was computed from EquiTest^®^ and hunova^®^ in both open (EO) and closed-eyes (EC) conditions. Results: Considering COP-related parameters, all correlations were significant in both EO (0.337 ≤ ρ ≤ 0.653) and EC (0.344 ≤ ρ ≤ 0.668). Furthermore, Pearson’s analysis of global balance indexes revealed relatively strong for visual and vestibular, and strong for somatosensory system associations (ρ = 0.573; ρ = 0.494; ρ = 0.710, respectively). Conclusions: Findings confirm the use of hunova^®^ as a valid device for dynamic balance assessment in MS, suggesting that such a robotic platform could allow for a more sensitive assessment of balance over time, and thus a better evaluation of the effectiveness of personalized treatment, thereby improving evidence-based clinical practice.

## 1. Introduction

Balance impairment is a major issue in Multiple Sclerosis (MS) leading to an increased risk of falling [1] that often prevents people with MS (PwMS) from performing their daily living activities [2,3], with a negative impact on their quality of life [4]. When compared to healthy controls of similar age, PwMS experience more falls as a result of these impairments [5]. Indeed, a meta-analysis on falls in MS showed that 56% of PwMS fall at least once per 3 months [6]. Central nervous system damage, observed in PwMS, leads to an altered central sensory integration of signals from muscle, tendon, joint proprioceptors, skin exteroceptors, and vestibular and visual inputs affecting postural response to maintain correct balance [7]. As indicated by Cameron and Nilsagård (2013), PwMS often present three main balance control abnormalities: decreased ability to maintain position when attempting to stand still; limited and slowed movement toward their limits of stability when attempting to lean or reach; and delayed automatic postural responses when displaced or perturbed [8]. Therefore, an accurate balance assessment is highly recommended for clinical practice and research as a key factor to monitor disease progression and tailor rehabilitative interventions. Extensive research has examined the contributions of physiological impairments to static balance [9,10,11,12,13], but falls most frequently occur during dynamic activities, such as walking or when surfaces are unstable [14,15]. The importance of assessing dynamic balance has been further highlighted by Anastasi and colleagues (2023): maintaining a stable gait requires high levels of motor control to integrate sensorimotor information about the position and the velocity of the Center of Mass (CoM); stabilize and redirect the CoM and provide an adequate foot placement at each step [1]. To this aim, several dynamic balance assessment tools have been developed, including clinical scales, such as the Fullerton Advanced Balance Scale (FAB) [16]. However, this scale has been shown to be suitable mainly for PwMS with low disability [17]. Other measures such as the Time Up and Go (TUG) [18] and the modified Dynamic Gait Index (DGI) [19] are common tests used to qualitatively and quantitatively assess instability during walking in PwMS, although they suffer from partial subjectivity, and poor sensitivity to change [1]. Given the need for a high-quality (precise, reliable, and valid) assessment of postural dynamic stability, additional balance assessment devices can be helpful in MS clinical settings. To overcome the aforementioned limitations of clinical scales and tests, posturography can provide valuable information about the individual’s postural stability, including the ability to maintain balance in different sensory conditions [12]. Kasser and colleagues (2011) [15] also demonstrated that reduced limits of stability on voluntary movement during dynamic posturography accurately identified frequent fallers in a sample of women with MS. However, to date, one of the gold standards in dynamic posturography, EquiTest^®^ (NeuroCom International, Inc., Clackamas, OR, USA) [20], presents some boundaries that limit its use in a clinical context (e.g., its reliance is limited only on an antero-posterior plane) [21]. Thus, in recent years, an increased number of new technologies, such as robotic-assistive systems, have been developed to provide a precise and complete assessment of balance in the neurological population, thanks to novel computational approaches as well as sophisticated electronic components [22]. Thus, investigating the correlation between conventional technology-driven performance assessments and innovative robotic methods for evaluating balance could be of utmost relevance in both MS clinical practice and research [23]. A recent study by Podda and colleagues (2023) confirms that a robotic platform can constitute an important innovative adjunct to balance assessment for PwMS [10]. In this study, authors compared the postural assessment during static tasks performed with an advanced robotic system and a standard posturography in a standing position [10]. Results indicated that robotic and standard outcomes strongly correlated considering Center Of Pressure (COP)-balance parameters and composite indexes calculated as global measures of balance. However, static balance assessment is usually performed in circumstances that are somewhat away from those encountered in daily-life scenarios. Since dynamic conditions, known to be more challenging than static ones, could provide more meaningful and ecological information on balance in MS, the aim of the present study was to investigate whether the postural assessment during dynamic tasks performed with hunova^®^ (Movendo Technology, SRL. Genoa, Italy) was comparable with the gold standard EquiTest^®^ [24] (NeuroCom International, Inc., Clackamas, OR, USA in a sample of PwMS.

## 2. Materials and Methods

### 2.1. Balance Assessment

**Figure 1:**
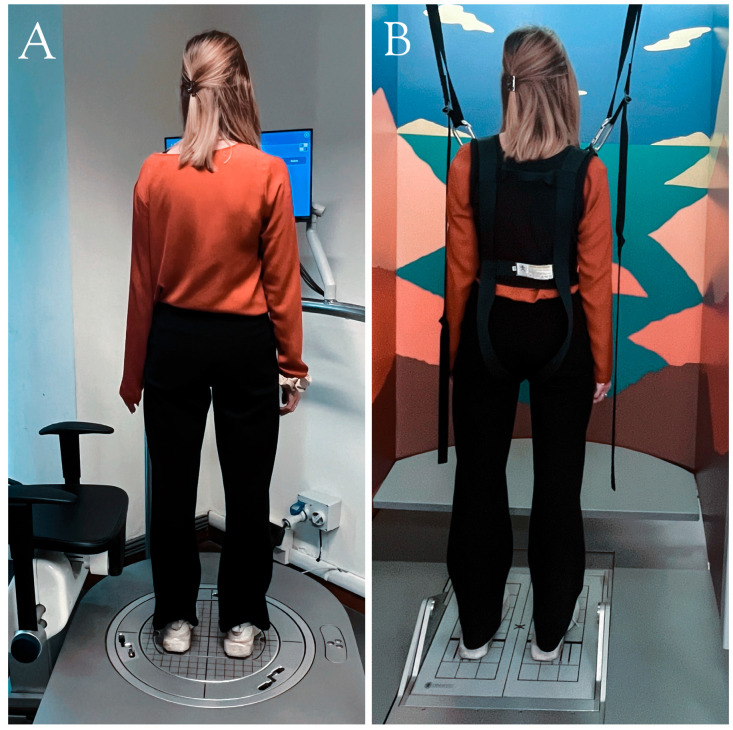
Devices used for the balance assessment of PwMS. (**A**) hunova^®^ from Movendo Technology S.R.L., Genoa, IT. hunova^®^ is a medical robotic device aimed at giving a response to the clinical need for the functional sensory–motor evaluation and rehabilitation of the ankle, lower limbs, and trunk that supports doctors, physiotherapists, and patients throughout assessments, treatments, and recoveries. This device enables the evaluation of balance while standing (both in mono- and bi-podalic configurations) and while sitting, both in static and dynamic testing conditions. (**B**) EquiTest^®^ from NeuroCom International, Inc., Clackamas, OR, USA. EquiTest^®^ allows the execution of standardized assessment protocols such as the Sensory Organization Test (SOT), Motor Control Test (MCT), and Adaptation Test (ADT) that are standard protocols for the assessment of balance disorders, dizziness, and mobility problems such as in MS.

Balance assessment was performed with both the EquiTest^®^ and hunova^®^ (See Figure 1). In particular, the sensory organization test (SOT) from EquiTest^®^ [24] and the Balance Test (BT) from hunova^®^ [25] were administered to measure balance (please see Supplementary Materials for an overview of both devices and their assessments).

The SOT is a six-condition assessment able to isolate and quantify impairments in the patient’s use of somatosensory, visual, and vestibular inputs to maintain balance, and impairments related to the patient’s use of specific sensory input when it is incorrect or unavailable [24]. In conditions 1 and 2 (COND 1 and COND 2), the participant stands quietly with eyes open (EO) and closed (EC), respectively; in both conditions, the platform and visual surround are fixed. In condition 3 (COND 3), the participant stands with EO; the platform is fixed, and the visual surround is sway-referenced (i.e., sway referencing refers to movement of the platform and/or visual surround in response to the body’s own sway, typically in an anterior-posterior direction). In condition 4 (COND 4), performed with EO, the support surface is sway-referenced whereas the surround is fixed. Condition 5 (COND 5) is performed with EC and a sway-referenced support surface, while the surround is fixed. Finally, in condition 6 (COND 6), the participant stands with EO; the visual surround and support surface are both sway-referenced. Each condition consists of three trials of 20 s. Scores are reported out of a maximum score of 100, indicating perfect balance and absence of sway. In addition to scores reported for each condition, there is also a composite score that represents all scores, with heavier weighting on those conditions that rely more heavily on the use of vestibular cues alone. Performances are evaluated using the Equilibrium Score that quantifies postural stability during each of the three trials of the six SOT conditions (see [10] for details on the computation).

**Figure 2:**
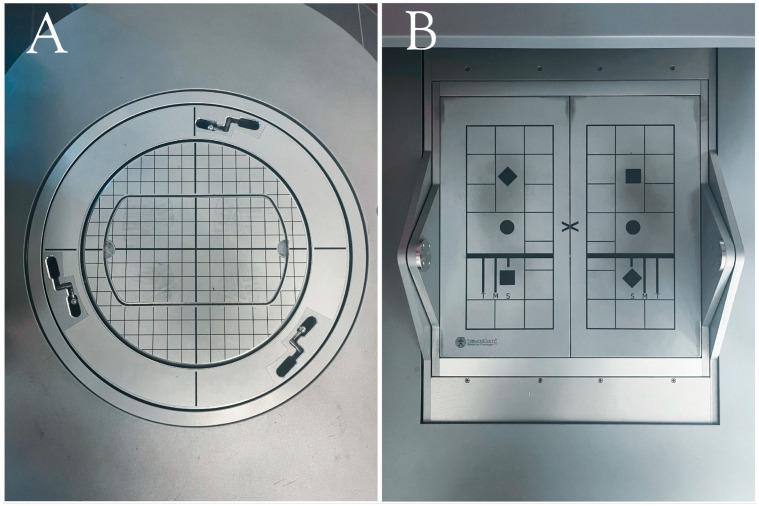
*hunova^®^* (**A**) and EquiTest^®^ platforms (**B**).

Designed as a robotic aid for healthcare professionals that is intuitive and easy to use, the use of hunova^®^ in different clinical settings such as neurology, orthopedics, and geriatrics is enlarging and has been validated in several studies and clinical trials with promising results [26,27,28,29]. BT on hunova^®^ allows testing balance under different conditions. More precisely, the device can simulate a static environment, or it can operate in a passive, an active, and an assistive modality. In the passive modality, the movements of the platforms are pre-planned following given trajectories with different speed levels. In the active modality, the user can actively move the platforms while it exerts a certain selectable resistance. When the assistive modality is selected, the device completes the exercise when subjects are unable to do it independently [30]. First, participants’ static balance was assessed with both devices in order to confirm previous results [10]. Static balance was tested with COND 1 and COND 2 of SOT for EquiTest^®^ and with two trials of BT performed with hunova^®^ on a static platform (s-BT) with both EO and EC. Then, dynamic balance was assessed using the COND 4 and COND 5 of the SOT and two trials of the elastic task of the BT (e-BT) performed on the unstable platform of hunova^®^ with both EO and EC. In e-BT, PwMS were asked to stand still on the unstable surface, which tilted in response to the weight shift of the subject. The platform responded as a plate on a pivot, with an additional low elastic rotatory force field that opposed the movement induced by the subject weight shift and tended to restore the platform parallel to the floor [25]. Figure 2 shows platforms from each device.

**Figure 3:**
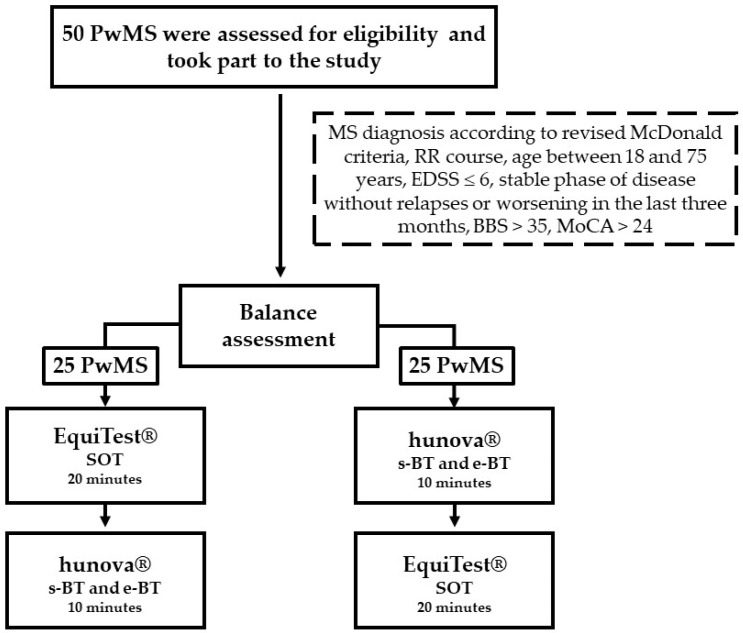
Flow chart of the study.

In both EquiTest^®^ and hunova^®^, participants were required to stand upright, with their arms relaxed along the sides of the body, looking straight ahead and avoid moving their feet for the entire duration of the test. The duration of the entire balance assessment was 30 min (EquiTest^®^: 20 min, hunova^®^: 10 min). The order of assessment delivered with EquiTest^®^ and hunova^®^ was counterbalanced between participants (see Figure 3 for a flow chart of the study).

#### 2.1.1. COP-Related Balance Measures

For both static and dynamic assessments, the following COP-related balance measures were calculated: sway area (SA) (cm^2^), Medio-Lateral (ML) and Anterior-Posterior (AP) oscillation range (cm), total path length (cm), ML and AP speed (cm/s), ML and AP root mean squared (RMS) distance (cm) [11]. While hunova^®^ easily provides these measures to the user, the same outcomes were not directly available for the EquiTest^®^ and thus were computed from raw data. All these measures are proportional to the instability of the individuals: the greater the values, the lesser the individual capability to maintain balance.

#### 2.1.2. Global Balance Indexes

From the average of the Equilibrium Scores of each condition, the sensory analysis was run to compute balance global scores such as SOM, calculated as COND 2/COND 1 (if under the normative threshold, it suggests the presence of a somatosensory impairment); VIS, as COND 4/COND 1 (if under the normative threshold, it suggests the inability to use vision for compensatory purposes); and VEST, as COND 5/COND 1 (if under the normative threshold, it suggests a possible vestibular deficit).

Although, by default, hunova^®^ does not directly compute the Equilibrium Score and, consequently, does not perform the sensory analysis, the aforementioned global parameters (SOM, VIS, and VEST) were calculated from the SA values. More precisely, the following equations were computed:

$${SOM}_{BT}=\frac{{SA}_{EC}\;of\;s-BT}{{SA}_{EO}\;of\;s-BT}$$

SOM represents the ratio of the SA of both EC and EO for s-BT;

$${VIS}_{BT}=\frac{{SA}_{EO}\;of\;e-BT}{{SA}_{EO}\;of\;s-BT}$$

VIS is the ratio of the SA of EO of e-BT to the SA of EO of s-BT;

$${VEST}_{BT}=\frac{{SA}_{EC}\;of\;e-BT}{{SA}_{EO}\;of\;s-BT}$$

Finally, VEST reflects the ratio of the SA of EC of e-BT to the SA of EO of s-BT.

### 2.2. Participants

PwMS were enrolled from those followed as outpatients at the AISM Rehabilitation Service of Genoa (Italy). Inclusion criteria were MS diagnosis according to revised McDonald criteria [31], age between 18 and 75 years, relapsing-remitting (RR) course, a disability level as measured by the Expanded Disability Status Scale (EDSS) [32] ≤6, stable phase of disease without relapses or worsening in the last three months, Berg Balance Score (BBS) [33] score > 35 indicating ability to stand upright and walking with at least one support, and normal cognitive functioning as indicated by a Montreal Cognitive Assessment (MoCA) [34,35] score ≥ 24. We excluded participants with psychiatric disorders, significant visual impairment defined as a Visual System scoring more than 2 at the Functional Systems Score of EDSS, and cardiovascular and/or respiratory disorders. Study procedures and consent forms conformed to the ethical standards of the 2013 revised Declaration of Helsinki and were approved by the regional ethical committee (Comitato Etico Regionale (CER) Liguria, reference number: 36/2022—DB id 12144). All the participants provided written informed consent to participate in the study and to state their agreement with the publication and communication of the results.

### 2.3. Statistical Analysis

To analyze sample demographic and clinical characteristics, main descriptive statistics (mean, standard deviation) were calculated. Data analysis was based on the raw data recorded during the static and dynamic trials executed with EquiTest^®^ and hunova^®^. Pearson’s ρ correlation was computed for each COP-related outcome and global balance index from EquiTest^®^ and hunova^®^. Correlation coefficients ranging from 0.20 to 0.39 were considered as moderate, from 0.40 to 0.59 as relatively strong, from 0.60 to 0.79 as strong, and higher as very strong correlation [36,37]. All *p* values were two-tailed and statistical significance was defined by alpha error < 0.05. All the algorithms for the calculus of the outcome measures were performed using MATLAB (MathWorks, Natick, MA, USA). Statistical analysis was performed with IBM SPSS Statistics software, 23.0.

## 3. Results

A total of 50 PwMS (32 females; mean age 52.9 ± 10.44 years; height: 166.76 ± 10.48 cm) were recruited for the study. Clinical characteristics showed a mean EDSS of 3.92 ± 1.38 and a mean disease duration of 12.22 ± 8.79 years. The BBS score was 49.49 ± 5.27 and the Ambulation Index score was 8.19 ± 3.64.

### 3.1. COP-Related Balance Measures Correlation

**Figure 4:**
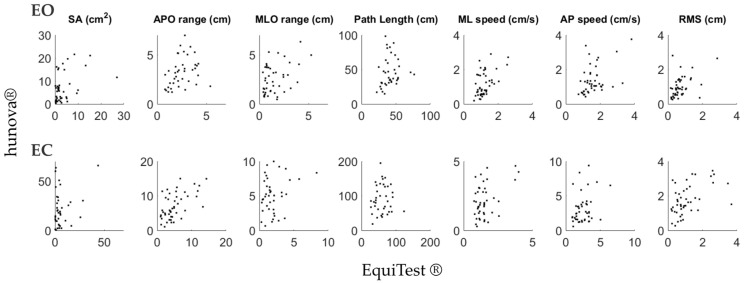
Graphical representation comparing COP-related parameters computed using EquiTest^®^ (*x*-axis) and hunova^®^ (*y*-axis) during static balance assessment. First raw: eyes open (EO); second raw: eyes closed (EC).

Table 1 presents mean values and relative standard deviations for each parameter computed for both EquiTest^®^ and hunova^®^, as well as the results of the correlation analysis on static balance data (See Figure 4 for a graphical representation of results). All correlations were significantly strong and relatively strong for both EO and EC conditions; only the APO range with EO was moderate. Findings confirmed previous results from [10].

**Figure 5:**
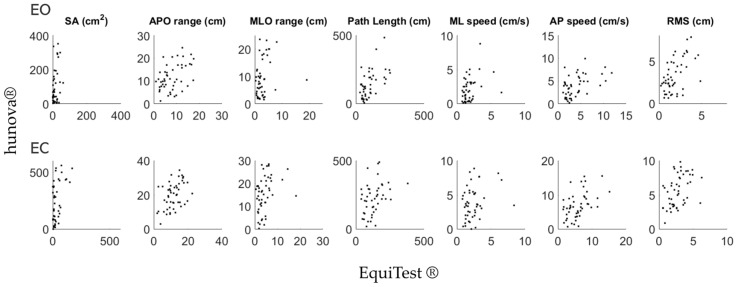
Graphical representation comparing COP-related parameters computed with EquiTest^®^ (*x*-axis) and hunova^®^ (*y*-axis) during dynamic balance assessment. First raw: eyes open (EO); second raw: eyes closed (EC).

Concerning dynamic tasks, correlations between the two devices aligned with those found for static balance (Table 2). All the correlations were significant and relatively strong or strong for both EO and EC conditions; only the MLO range with both EO and EC was moderate (See Figure 5 for a graphical representation of results).

### 3.2. Global Balance Indexes Correlation

The results of the correlations between devices on global balance revealed relatively strong correlations for VIS and VEST (ρ = 0.573 and ρ = 0.494, respectively), and strong for SOM (ρ = 0.710) (Table 3).

## 4. Discussion

In this study, we compared the dynamic balance assessment performed with hunova^®^, a robotic medical device, and EquiTest^®^, the gold standard for postural assessment, in a sample of PwMS. Globally, our findings demonstrate the use of hunova^®^ as a valid and reliable device for balance assessment in MS, as well as for dynamic tasks. Here, as a first step towards the aim, we confirmed results from the previous study by Podda and colleagues [10] that found that hunova^®^ measures were highly comparable to those from EquiTest^®^ for static balance in PwMS. Then, considering dynamic balance, the COP-related measures extracted from the raw data significantly correlated, showing moderate to strong associations in both EO (0.337 ≤ ρ ≤ 0.653) and EC (0.344 ≤ ρ ≤ 0.668). In addition, the correlation analysis on global balance indexes led to remarkable results for SOM (ρ = 0.710), VIS (ρ = 0.573), and VEST (ρ = 0.494). This is in line with Tacchino and colleagues [38], who recently demonstrated that both VEST and VIS systems correlated with the Six-Spot-Step-Test, a timed measure of the participant’s ability to maintain balance while challenging their stability during walking [39]. Authors suggest that people with problems in the organization and integration of information from multiple sensory systems involved in balance maintenance could have difficulty in safely navigating through environments with conflicting visual surroundings information, surface changes/irregularities, or obstacles on the ground. In our study, while the strong association for the SOM was expected, as this metric compared the static performance in EC and EO, the other strong correlations were unexpected, given that VIS and VEST indexes may reflect also structural and mechanical differences between the two devices. Indeed, COND 4 and COND 5 of the SOT from the EquiTest^®^ and the e-BT of hunova^®^ are not exactly calculated in the same way. Despite in these conditions the platform of each device moves accordingly to the subject’s sway shift, with movements proportional to the oscillation of the subject, the support surface of the EquiTest^®^ moves only in the AP direction, while hunova^®^ allows for movements in all directions (both AP, ML, and a combination of the two). This is further confirmed by the results from COP-related measures, in which APO and MLO correlations in EO (ρ = 0.506; ρ = 0.337) reflected differences in the ranges of maximum oscillations of the two devices. Dynamic balance disorders are more evident in the ML than in the AP direction, in accordance with the view that ML stabilization is critical for walking [1] and that any deficit in ML postural control is more alarming in MS given that falls to the side are more likely to cause hip fractures [40]. Healthy adults often compensate for a loss of balance due to unexpected ML perturbations with a variety of strategies, such as a lateral ankle strategy (i.e., using an inversion moment that quickly moves the center of pressure to the outer limit of the foot to compensate for medial perturbations) or a hip strategy (i.e., a hip abduction moment to assist in maintaining balance by counteracting the gravitational moment) [41]. As indicated by Anastasi and colleagues (2023) [1], the Margin of Stability (MoS) in the ML direction may be more sensitive than MoS in AP in describing balance disorders in PwMS with moderate to severe disability. Higher ML sway may reflect a “degrees of freedom” problem [40]: with fewer muscles involved in controlling ML motion compared to AP, PwMS may have greater difficulty in adequately activating the hip abductor/adductor muscles to maintain balance [42]. Therefore, it can be reasonable to conclude that e-BT from hunova^®^ is able to simulate common postural disturbances that PwMS experience in their lives (e.g., standing on a bus, slipping on a slippery surface, and falling due to a sudden boost), while EquiTest^®^, missing perturbations in ML direction, only gave an incomplete view within balance assessment of PwMS. This suggests that a robotic device such as hunova^®^ that considers both AP and ML directions (and its combination) should be recommended since detailed clinical testing may give important guidance in the prevention and rehabilitation of balance disorders in PwMS.

Our findings provide further support for the use of such robotic devices as additional or even in substitution for standard posturography in the MS population. Other than reliance only on AP direction, the safety harness in EquiTest^®^, fixed to the safety bar, which prevents users from falling, tends to independently stabilize participants’ posture as their COG approaches the limits of stability [10].

In discussing our data, some important limitations need to be considered. Participants’ characteristics may limit the interpretation of our results. The study sample was constituted of individuals able to walk with at least one walking aid (EDSS ≤ 6). Therefore, results may not generalize to individuals with MS with a higher disability level who require two walking aids such as a pair of canes or crutches (EDSS = 6.5). In addition, future studies should evaluate the responsiveness or sensitivity to balance change, investigating the potential of hunova^®^ as an integrated device for both assessment and rehabilitation purposes for PwMS.

To conclude, hunova^®^ could allow for a more sensitive assessment of dynamic balance over time as the disease progresses, and thus a better evaluation of the effectiveness of tailored treatment for everyone, thereby improving evidence-based clinical practice in MS. Furthermore, our findings suggested the importance of a detailed assessment of dynamic balance through sophisticated robotic devices able to closely resemble daily living challenging activities, such as standing or walking on instable surfaces.

## Figures and Tables

**Table 1:** Results of the Pearson’s ρ correlations between COP-related balance measures from EquiTest^®^ and hunova^®^ in EO and EC conditions during static tasks.

		EquiTest^®^		hunova^®^			
		Mean	STD	Mean	STD	Pearson	*p*
EO	SA (cm^2^) APO range (cm) MLO range (cm)	5.29	11.61	10.07	12.39	0.596	0.000
		3.09	2.22	4.01	2.56	0.355	0.013
		1.73	1.63	3.04	2.14	0.520	0.000
	Path Length (cm)	47.02	21.08	57.96	35.23	0.474	0.001
	ML speed (cm/s)	1.20	0.42	1.15	0.69	0.510	0.000
	AP speed (cm/s)	1.74	0.93	1.54	1.03	0.459	0.001
	RMS (cm)	0.71	0.51	0.97	0.59	0.457	0.001
EC	SA (cm^2^) APO range (cm) MLO range (cm)	16.15	33.71	32.30	29.43	0.572	0.000
		5.15	3.38	7.09	3.73	0.574	0.000
		2.69	2.83	5.76	2.97	0.451	0.001
	Path Length (cm)	76.35	56.03	127.82	88.93	0.686	0.000
	ML speed (cm/s)	1.60	0.89	2.25	1.31	0.540	0.000
	AP speed (cm/s)	3.08	2.52	3.65	2.83	0.722	0.000
	RMS (cm)	1.19	0.98	1.78	0.88	0.553	0.000

**Table 2:** Results of the Pearson’s ρ correlations between COP-related balance measures from EquiTest^®^ and hunova^®^ in EO and EC conditions during dynamic tasks.

		EquiTest^®^		hunova^®^			
		Mean	STD	Mean	STD	Pearson	*p*
EO	SA (cm^2^) APO range (cm) MLO range (cm)	16.52	17.26	112.59	129.85	0.498	0.000
		8.34	4.77	12.07	6.49	0.506	0.000
		3.07	3.13	9.72	7.11	0.337	0.018
	Path length (cm)	106.73	79.32	164.67	154.40	0.653	0.000
	ML speed (cm/s)	1.89	1.22	2.22	2.18	0.533	0.000
	AP speed (cm/s)	4.53	3.69	5.00	4.97	0.622	0.000
	RMS (cm)	1.85	1.13	3.26	1.90	0.561	0.000
EC	SA (cm^2^) APO range (cm) MLO range (cm)	39.43	38.21	309.91	229.96	0.413	0.004
		11.63	4.72	19.80	7.56	0.436	0.002
		4.34	3.40	15.43	7.68	0.344	0.016
	Path length (cm)	146.36	83.32	257.18	158.51	0.621	0.000
	ML speed (cm/s)	2.49	1.54	3.95	2.61	0.403	0.004
	AP speed (cm/s)	6.32	3.69	7.56	4.83	0.668	0.000
	RMS (cm)	2.77	1.35	5.62	2.28	0.435	0.002

**Table 3:** Results of the Pearson’s ρ correlations between global balance measures from EquiTest^®^ and hunova^®^ in EO and EC conditions during dynamic tasks.

	EquiTest^®^	hunova^®^		
	Mean STD	Mean STD	Pearson	*p*
SOM	4.4 9.7	5.8 6.3	0.710	0.000
VIS	10.5 15.6	20.3 27.0	0.573	0.000
VEST	21.7 22.0	84.8 110.5	0.494	0.000

## Data Availability

Data will be available on demand, from the corresponding author. The data are not publicly available due to privacy reasons.

## Supplementary Materials

The following supporting information can be downloaded at: https://www.mdpi.com/article/10.3390/s24113325/s1. The supporting information includes an overview of EquiTest^®^ and hunova^®^ and their balance assessments.
