# Supplementary material for: Testing Dynamic Balance in People with Multiple Sclerosis: A Correlational Study between Standard Posturography and Robotic-Assistive Device
Source: Sensors (Basel). 2024 May 23;24(11):3325. doi: 10.3390/s24113325 (PMC11174503; doi:10.3390/s24113325)
Supplement: Supplementary file 1 [file sensors-24-03325-s001.zip › Podda et al_Supplementary Material_Sensors.pdf]

TITLE: Testing dynamic balance in people with Multiple Sclerosis: a correlational study between standard posturography and robotic-assistive device.

SUPPLEMENTARY MATERIAL:

EquiTest®

The EquiTest® consists of a movable 46 x 46 cm dual force plate, with two twin platforms connected by a pin joint oriented in the left–right direction, crossing the centre of the anterior–posterior axis. The two platforms can tilt simultaneously around the pin joint and glide in the anterior–posterior (AP) direction through a PC-controlled servomotor. Each EquiTest® plate has two sensors of vertical forces (A/D conversion: 100 Hz sampling rate, 12 bit) and a fifth transducer, sensitive to AP shearing forces, is bracketed to the centre plate directly beneath the pin joint.

EquiTest® allows the execution of standardized assessment protocols such as the Sensory Organization Test (SOT), Motor Control Test (MCT) and the Adaptation Test (ADT) that are standard protocols for the assessment of balance disorders, dizziness and mobility problems such as in MS[1]. The SOT is a six-condition assessment able to isolate and quantify impairments in the patient's use of somatosensory, visual, and vestibular inputs to balance, and impairments related to the patient's use of specific sensory input when it is incorrect. In conditions 1 and 2 (COND 1 and COND 2), the participant stands quietly with eyes open (EO) and closed (EC), respectively; in both conditions the platform and visual surround are fixed. This establishes whether sway increases when visual cues are removed and determines how effectively the participant makes use of somatosensory input. In condition 3 (COND 3), the participant stands with EO; the platform is fixed and the visual surround is sway-referenced and, for this reason, the visual cues become inaccurate. In condition 4 (COND 4), performed with EO, the support surface is sway-referenced whereas the surround is fixed; thus, somatosensory cues become inaccurate. Condition 5 (COND 5) is performed with EC and a sway-referenced support surface, while the surround is fixed. This determines how the participant makes use of vestibular cues when visual cues are removed, and somatosensory cues are inaccurate. Finally, in condition 6 (COND 6), the participant stands with EO; the visual surround and support surface are both sway-referenced, which identifies if the participant relies on visual cues even when they are inaccurate. Thus, it provides information about interactions among the three sensory systems contributing to postural control. The SOT also quantifies secondary maladaptive impairments related

to the patient's ability to select appropriate movement strategies and to accurately align their center of gravity (COG) relative to their base of support.

The SOT provides several analyses used to assess balance performances:

- Equilibrium score that quantifies postural stability during each of the three trials of six sensory conditions.
- Sensory analysis that reflects the sensory ratios computed from the average equilibrium scores in order to identify the subject's ability to use input to maintain balance from somatosensory system (COND-2/COND-1), visual system (COND-4/COND-1) and vestibular system (COND-5/COND-1). Moreover the sensory analysis allows to establish the degree to which the subject relies on visual information to maintain balance, even when the information is incorrect ((COND-3 + COND-6)/(COND-2 + COND-5)).
- Strategy analysis that quantifies the relative amount of movement about the ankles (ankle strategy) and the hips (hip strategy) used by the subject to maintain balance during each trial.
- Center of Gravity alignment that reflects the subject's center of gravity position relative to the support base center at the start of each SOT trial. People with normal performance maintain their center of gravity close to the center of the support base.

The MCT assesses the ability of the automatic motor system to quickly and effectively recover from unexpected support surface disturbances. The MCT isolates and quantifies impairments in the timing and strength of the automatic response in each leg; furthermore, it detects impairments in coordination of responses between the two legs and movement directions.

The ADT tests the automatic motor system and to quantify impairments in the subject's ability to adapt automatic responses to minimize sway when exposed to surface irregularities and unexpected changes in support surface inclination. It also quantifies the subject's capacity to systematically reduce their sway energy during repeated exposure to the same surface tilt disturbance.

The EquiTest® also assesses the Limits of Stability (LOS) that quantifies impairments in ability to intentionally displace the COG to the subject's stability limits without losing balance, the Rhythmic Weight Shift (RWS) that quantifies the subject's ability to perform rhythmic movements of their COG from left to right and forward to backward at three distinct paces, the Weight Bearing Squat (WBS) that quantifies the subject's ability to perform squats with the knees flexed at 0°, 30°, 60°, and 90°, while maintaining equal weight on the two legs, and the Unilateral Stance (US) that quantifies the

subject's ability to maintain postural stability while standing on one leg at a time with the eyes open and closed.

hunova®

hunova® is a new medical robotic device aimed at giving a response to the clinical need for the functional sensory–motor evaluation and rehabilitation of the ankle, lower limbs and trunk that support doctors, physiotherapists, and patients throughout assessments, treatments, and recoveries [3]. This device enables the evaluation of balance while standing (both in mono- and bi-podalic configurations) and while sitting, both in different testing conditions, such as static and dynamics; it has different difficulty levels for each task, and it integrates both visual and auditory feedback based on the user's performance. hunova® consists of two electromechanical and sensorized platforms with two degrees of freedom (forwards/backwards and left/right), one at the foot level and one at the seat level. The device operates in conjunction with a wireless 9-axis Inertial Movement Unit (IMU), including accelerometer, gyroscope and magnetometer to monitor trunk movements (i.e. located on the subject's torso). hunova® also comprises a tablet device which runs the clinician user interface. This graphical user interface (GUI) allows the operator to manage patients' database, single patient's training and assessment history and start exercise sessions directly from the tablet. The tablet is connected to the robotic system via a WiFi link [4].
As we mentioned before, hunova® allows testing balance under different conditions. More precisely, the device can simulate a static environment, or it can operate in a passive, an active and an assistive modality. In the passive modality, the movements of the platforms are pre-planned following given trajectories with different speed levels. In the active modality, the user can actively move the platforms while it exerts a certain selectable resistance. When the assistive modality is selected, the device completes the exercise when subjects are unable to do it independently.
Just to give a brief overview of the potentials of the device, in the following we list some of the trainings it enables:

- Balance tasks (both in mono- and bi-podalic configurations when standing, and while sitting)
    - on static platform
    - on unstable platform
    - on counter-resistance platform

    - on variable inclined plane
    - on a moving platform (following different trajectories)
    - on a platform giving impulsive perturbations
- Dual task: balancing on either static or moving platform while performing reaching tasks with the upper limbs (both in mono- and bi-podalic configurations when standing, and while sitting)
- Limits of stability tests (both in mono- and bi-podalic configurations when standing, and while sitting)
- Strengthening tasks, such as squats, isometric/isotonic and isokinetic tests
- Mobilization of the ankle
- Torso control
- Core stability tasks.

Reference

1. NeuroCom International NEUROCOM® INTERNATIONAL, INC. Balance Manager® Systems Technical Specifications. **2008**, *6744*, 1–8.
2. Vanicek, N.; King, S.A.; Gohil, R.; Chetter, I.C.; Coughlin, P.A. Computerized Dynamic Posturography for Postural Control Assessment in Patients with Intermittent Claudication. **2013**, 1–9, doi:10.3791/51077.
3. Movendo Technology Available online: https://www.movendo.technology/our-products/hunova/?lang=en.
4. Saglia, J.A.; Luca, A. De; Squeri, V.; Ciaccia, L.; Sanfilippo, C.; Ungaro, S.; Michieli, L. De Design and Development of a Novel Core, Balance and Lower Limb Rehabilitation Robot: Hunova®. *IEEE Int. Conf. Rehabil. Robot.* **2019**, *2019-June*, 417–422, doi:10.1109/ICORR.2019.8779531.
